# Para aortic ganglioneuroma presenting as Cushing's syndrome

**DOI:** 10.4103/0970-1591.36725

**Published:** 2007

**Authors:** Jaya Ram Reddy S., G. Purushottam, K. Pandurangarao, P. T. Ravi Chander

**Affiliations:** Department of Urology, Osmania General Hospital, Hyderabad, India

**Keywords:** Cushing's syndrome, ectopic ACTH, ganglioneuroma, para aortic

## Abstract

We present a case of an eight-year-old female presenting with four months history of progressive weight gain, short stature, obesity, mild acanthosis, moon facies and buffalo hump. Biochemically, low-dose and high-dose Dexamethasone tests were not suppressible, ACTH was raised and 24h urinary metanephrines were normal. The CECT scan showed a 3cm paraganglioma. Tumor was excised via 11^th^ rib transcostal approach and the mass was found arising from the sympathetic chain. Histopathology was suggestive of ganglioneuroma positive for ACTH immunostaining.

## INTRODUCTION

Association of Cushing's syndrome with ganglioneuroma is very rare. Ganglioneuromas may be hormonally active. Hypertension, diarrhea, flushing and virilization may occur as a result of the secretion of catecholamines, vasoactive intestinal polypeptide or androgenic hormones. This case is presented due to its extreme rarity.

## CASE REPORT

**Figure 1:**
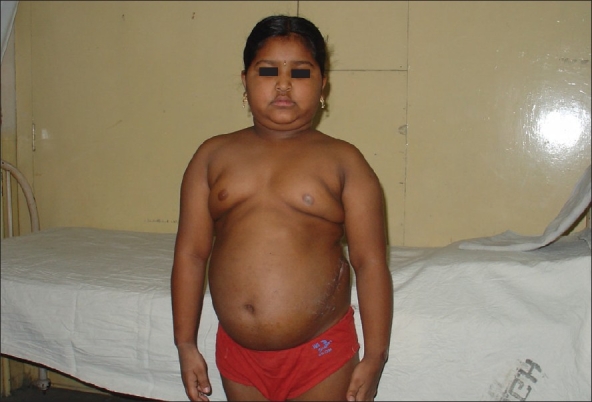
Clinical photograph of the patient. Operative scar can be seen

An eight-year-old female [Figure 1] presented with progressive weight gain since four months and increased appetite. There was no history of decreased visual acuity, diplopia or features suggestive of hypothyroidism. No proximal muscle weakness was noted. Her height was 127cm, weight 32kg (wt. age 9.5y) and BMI was 19.84. She has mild acanthosis, moon facies, double chin, buffalo hump and spinal tenderness over the lower thoracic spine. She had high blood pressure (170/90). Systemic examination was normal. Her hormonal profile [Table 1] showed an insuppressible LDDST and HDDST, high serum ACTH and normal 24h urinary metanephrines.

**Table 1:** Patient's hormonal profile

Test	Patient value	Normal value
LDDST (Low-Dose Dexamethasone Suppression Test)	2.99 microgram/dl	Less than 1.8 microgram/dL
HDDST (High-Dose Dexamethasone Suppression Test)	2.25 microgram/dl	Suppressed to less than 50% the LDDST suggesting ectopic or an adrenal source
Serum ACTH	90.4 pg/dL	Upper limit 70 pg/dL
24h urinary metanephrines	0.7 microgram/dl	Less than 1.0 microgram/dL

**Figure 2:**
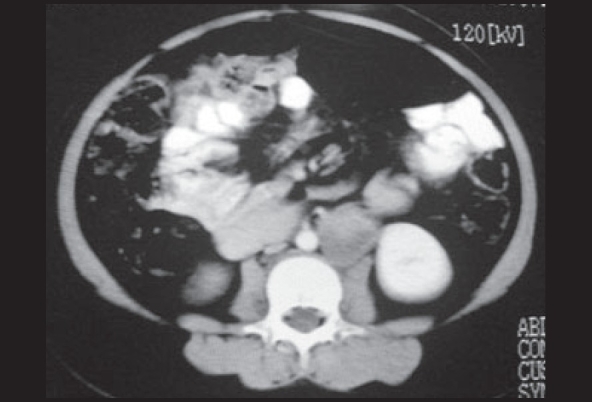
CECT abdomen showing a 3.2 × 2.6 × 6 cm dense mass lesion in the left para aortic region near the interpolar region of left kidney enhancing moderately with contrast

The CT scan abdomen [Figure 2] showed a dense mass lesion measuring 3.2 × 2.6 × 6 cm in the para aortic region arising at the interpolar region of the left kidney, had a speck of calcification. With IV contrast, tumor showed enhancement and arterial blush, suggestive of paraganglioma. Both the adrenals were normal.

**Figure 3:**
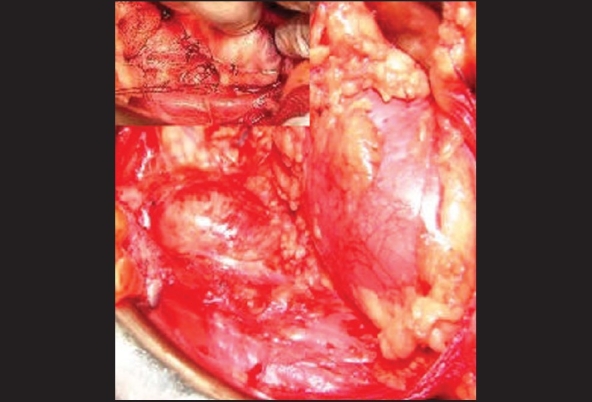
Operative photograph showing tumor in the para aortic region. Lower pole of the left kidney can be seen in the picture. Inset showing tumor with its attachmets to the sympathetic chain being divided

Through 11^th^ rib transcostal retroperitoneal approach a 6 × 4 cm tumor [Figure 3] at the level of L3 L4 adherent to sympathetic chain, abutting the aorta and psoas muscle with blood supply being derived directly from aorta was completely excised [Figure 3]. Postoperative period was uneventful.

**Figure 4:**
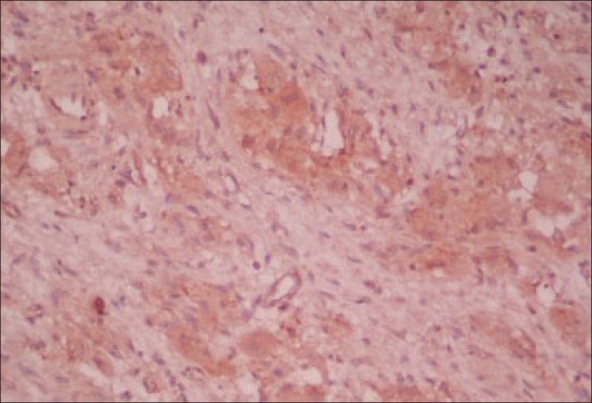
Histopathology showing ganglion cells scattered in small aggregates between the fascicles of spindle schwanian cells. Immunostaining positive for ACTH. This can be appreciated with the brown colour uptake of the cytoplasm in ganglion cells

Grossly this grayish white nodular mass was homogeneous on cut section. Microscopically tumor was encapsulated showing proliferation of spindle Schwannian cells in short fascicles. Ganglion cells were conspicuous in between, scattered in small aggregates [Figure 4]. Spherules of calcification were present. No neuroblastoma component could be identified. Features were in favor of a ganglioneuroma. Immunostaining for ACTH was positive [Figure 4].

Two lymph nodes included showed mild reactive changes with no evidence of metastases.

Final diagnosis was an ectopic ACTH-secreting para aortic ganglioneuroma Stage 1(INNS International Neuroblastoma staging system)[1] causing Cushing's syndrome.

### Follow-up

Follow-up period was six months. Clinically, the patient lost 6kg in weight, showed improvement in her daily performance. Biochemically, serum ACTH and cortisol levels were normal at the end of one month post op. Patient is off the antihypertensives. Monthly follow-up during the first postoperative year was planned to detect early recurrence if any.

## DISCUSSION

Ganglioneuromas originate from neural crest sympathogonia (undifferentiated cells of the sympathetic nervous system). Neuroblastomas, ganglioneuromas and ganglioneuroblastomas are collectively known as neuroblastic tumors.[2]

These tumors may show hormonal activity. Hypertension, diarrhea, flushing and virilization may occur as a result of the secretion of catecholamines, vasoactive intestinal polypeptide or androgenic hormone.[2] Though these tumors have the machinery to produce any peptide hormone, production of other than catecholamines, vasoactive intestinal polypeptide or androgenic hormones is very rare. Occasional reports of ACTH/CRH-producing ganglioneuromas presenting clinically as Cushing's syndrome were reported.

Most frequently these tumors occur in the abdomen. Common locations include the adrenal gland, paraspinal retroperitoneum (sympathetic ganglia), posterior mediastinum, head and neck. Locations such as the urinary bladder, bowel wall, abdominal wall and gallbladder were considered unusual.

Ganglioneuromas are rare, benign, fully differentiated tumors that contain mature Schwann cells, ganglion cells, fibrous tissue and nerve fibers.[3] These tumors do not show immature elements (such as neuroblasts), atypia, mitotic figures, intermediate cells or necrosis. These tumors can arise de novo or result from the maturation of a ganglioneuroblastoma or a neuroblastoma into a ganglioneuroma.[3]

Ganglioneuromas are staged using the INSS (International Neuroblastoma Staging System).[3] Treatment is usually in the form of surgical excision for localized non metastatic tumors. Overall, patients with ganglioneuroma have a favorable prognosis.

Cushing's syndrome due to ectopic ACTH production poses diagnostic challenges by mimicking the pituitary-dependent form of Cushing's syndrome. Ectopic ACTH syndrome has a slight female predominance. Bronchial carcinoid, Islet cell cancer, Small cell carcinoma lung, medullary thyroid carcinoma were among the common etiologies.[4,5]

The first case report of ectopic ACTH syndrome induced by an extra adrenal abdominal paraganglioma (pheochromocytoma) has been reported in 2005.[6] The present case is unique as this is an ectopic ACTH-producing para aortic ganglioneuroma causing Cushing's syndrome, first of its kind to be reported.
